# Invasive *Rosa rugosa* populations outperform native populations, but some populations have greater invasive potential than others

**DOI:** 10.1038/s41598-018-23974-3

**Published:** 2018-04-10

**Authors:** Shuping Zhang, Maike Isermann, Wenhao Gan, Martin Breed

**Affiliations:** 1 Institute of Ecology and Biodiversity, School of Life Sciences, Shandong University, 250100 Jinan, China; 2 Department of Plant Ecology and Evolution, Evolutionary Biology Centre, Uppsala University, SE-75236 Uppsala, Sweden; 3 Vegetation Ecology and Conservation Biology, Department of Ecology, FB 2, Bremen University, D-28359 Bremen, Germany; 4 School of Biological Sciences, University of Adelaide, South Australia, 5005 Australia

## Abstract

Increased performance of invasive plant species in their introduced range vs. their native range has been previously documented. However, performance differences among invasive populations have rarely been explored, despite this information being central to understanding the evolution of invasiveness as well as being a useful basis to inform management of invasive species. To examine variation in performance among populations of *Rosa rugosa* in its introduced range, and whether introduced populations perform better than native populations, we quantified growth and reproductive traits in five invasive populations in northwest Europe and two native and declining populations in China. Overall, we found that the introduced *R. rugosa* populations we sampled performed significantly better than the sampled native populations for growth and reproductive traits (2 to 4 fold increase). However, there was significant variation for most traits among the five invasive populations, demonstrating that some introduced populations we sampled were more successful invaders than others. Our findings provide a useful foundation for management of invasive *R. rugosa* in Europe, and support the recent call for more intra-species research in invasive species biology.

## Introduction

Increased performance of invasive plants in their invasive range has been well documented^1,2^, with many performance comparisons between invasive and native species pairs^3^. However, few studies have compared performance differences among invasive populations^4,5^, which is surprising since quantifying the relative performance of different populations should indicate their invasive potential. Consequently, studies that compare performance differences among invasive populations will help to identify populations that present a higher risk of becoming invasive, and this evidence should be used to guide management of emerging and existing invasive species^6^.

A number of growth and reproduction traits have been associated with invasiveness, including plant height, biomass and reproductive output^3,7^. For example, high relative growth rate and reproductive output were associated with greater invasiveness in dayflowers and multiflora rose (*Commelina benghalensis* - Commelinaceae; *Rosa multiflora*)^8,9^. However, the traits that correlate with invasiveness have been frequently found to be species or even population specific^10^, which may be because too few studies have been done to identify general trends, or that a large number of traits may contribute to invasiveness. Therefore, more research on identifying traits that correlate with invasiveness is necessary, and particularly in species that are potent invaders or those that display considerable heterogeneity in invasiveness between populations.

Most previous studies on performance differences between invasive and native populations have been done on short-lived plants (e.g. herbs, grasses) or on the early life stages of woody species^11–13^. Consequently, traits such as plant size, height and reproduction output have rarely been studied in woody invasive plants. This is despite the fact that studying these traits in woody plants is important because many shrubs and trees are major invasive species^14^. A pragmatic solution to this problem is to collect trait data *in situ*^9,15^. Indeed, traits measured *in situ* have been important for studying performance differences between native and invasive populations of several shrub species, such as *Buddleja davidii* and *Rosa rubiginosa*^5,16^.

In this study, we quantified performance traits in a number of invasive and native populations of the shrub *Rosa rugosa*. Its native range includes China, the Korean Peninsula, Japan and northwards to the Kamchatka peninsula, where it is sensitive to environment change, particularly habitat loss due to human building activities^17–20^. It has been listed as an endangered species in China due to a rapid decline in the number of populations^21–23^. However, it has been introduced and subsequently become naturalized on sandy beaches and sand dunes along the Baltic Sea and North Sea in northwest Europe. It was first introduced into England from Japan in 1796, and the first naturalized record of *R. rugosa* in continental Europe was in Germany in 1845, then in Denmark in 1875, and Sweden in 1918. In 2001, 16 European countries had records of naturalized *R. rugosa* populations^24^.

*Rosa rugosa* is an appropriate model for studying invasiveness because the invasive European populations and the native Chinese populations present two extreme situations of population dynamics. In Northwest Europe, *R. rugosa* often forms dominant, large, and dense scrub that excludes, at a local scale, native species^25,26^. Unlike many invasive species that harbour lower genetic diversity in their invasive range than their native range^27–29^, European *R. rugosa* populations show similar levels of expected heterozygosity (*H*_*e*_) as native populations in the Far East of Russia, East Asia and northern Japan^30,31^.

We studied five naturalized invasive *R. rugosa* populations in northwest Europe and two native populations in China to explore patterns of performance trait differentiation between native and invasive populations. We focused on the following questions: (1) Whether our sampled introduced *R. rugosa* populations perform better than the sampled native populations? (2) How heterogeneous is the performance of the sampled introduced populations? (3) Which traits contributed to the performance differences among the sampled introduced populations? By answering these questions, we identify which introduced populations are most invasive and which traits are indicative of this invasiveness. Furthermore, we discuss possible factors driving performance differentiation during invasion.

## Results

### Regional differences

At a regional scale, the sampled *Rosa rugosa* population in Europe outperformed the sampled *R. rugosa* population in native China, with the European population showing significantly greater values in all traits except the ratio of hip length to width (Tables 1 and 2). Of the reproductive traits, mean hips/m^2^, hip volume, and seeds/hip were 2.7, 2.3 and 1.9 times higher in Europe than in China. This resulted in a 4.2 times higher value of mean seeds/m^2^ in Europe than in China. Among the growth traits, mean shrub size, percentage cover and mean shrub height were 3.2, 1.7 and 1.5 times higher in Europe than in China. For hip length: width, Europe had significantly flatter hips (smaller ratio of hip length to width) than China.

**Table 1:** Nested ANOVA results showing effects of region and population nested within region on eight performance traits of *Rosa rugosa*.

Trait	Factor	df	SS	MS	F	P
**Reproductive traits**						
Hips/m^2 a^	Region	1	3328.00	3328.20	5.81	<0.05*
	Region: population	4	12760.00	3189.90	5.56	<0.001***
	Residual	65	37263.00	573.30		
Seeds per hip^a^	Region	1	5967.90	5967.90	21.27	<0.001***
	Region: population	4	1639.60	409.90	1.46	0.224 NS
	Residual	65	18240.30	280.60		
Seeds/m^2 a,b^	Region	1	3189.00	3189.00	11.81	<0.001**
	Region: population	4	5824.10	1456.00	5.39	<0.001***
	Residual	65	17554.20	270.10		
Hip length: width	Region	1	0.41	0.41	55.48	<0.001***
	Region: population	4	0.13	0.03	4.34	<0.01**
	Residual	65	0.48	0.00		
Hip volume (cm^3^)	Region	1	31.816	32.82	30.39	<0.001***
	Region: population	4	50.92	12.73	12.16	<0.001***
	Residual	65	68.05	1.05		
**Growth traits**						
Shrub size (m^2^)^b^	Region	1	740.02	740.02	10.47	<0.01**
	Region: population	5	962.90	192.58	2.72	<0.05*
	Residual	74	5232.00	70.70		
Cover (%)	Region	1	14931.00	14930.00	50.85	<0.001***
	Region: population	5	4249.70	849.90	2.89	<0.05*
	Residual	74	21727.30	293.60		
Height (cm)^c^	Region	1	13912.50	13912.50	20.97	<0.001***
	Region: population	5	11490.00	2298.00	3.46	<0.01**
	Residual	56	37161.00	663.60		

Significant P values were shown as follows: ‘***’ < 0.001, ‘**’ < 0.01, ‘*’ < 0.05. df is degrees of freedom, SS is sum of squares, MS is mean squares, F is the F statistic.

^a^Traits without data from population HCH, since there was no available hip and seed data due to flower collection by local residents.

^b^Traits were square root transformed before ANOVA to increase normality and decrease heteroscedasticity of the variables.

^c^Trait was analyzed after omitting 18 shrubs with mowing management in HUN, DRA, HEA, SKA.

**Table 2:** Performance traits comparison of *Rosa rugosa* between introduced European range and native Chinese range.

Trait	Introduced Europe				Native China			
	n	Mean	Range	SD	n	Mean	Range	SD
**Reproductive traits**								
Hips/m^2^	61	32	1–112	28	10	12	2–36	11
Seeds/hip	61	58	20–101	18	10	31	24–43	5
Seeds/m^2^	61	1862	46–7171	1735	10	440	76–1044	354
Hip volume (cm^3^)	61	3.41	0.83–5.94	1.40	10	1.48	1.08–2.19	0.40
Hip length: width	61	0.72	0.59–1.02	0.09	10	0.94	0.80–1.18	0.11
**Growth traits**								
Shrub size (m^2^)	61	368	3.00–3000.00	493.65	20	113.25	8.00–520	117.47
Cover (%)	61	75	23.00–98.00	18.05	20	43.40	10–72	18.39
Height (cm)*	43	97	47.00–177.00	31.40	20	64.75	34.0–103.0	19.53

Values significantly smaller in the introduced European range than in the native Chinese range are underlined.

*Trait was analyzed after omitting all 18 shrubs with mowing management in HUN, DRA, HEA, SKA.

**Figure 1:**
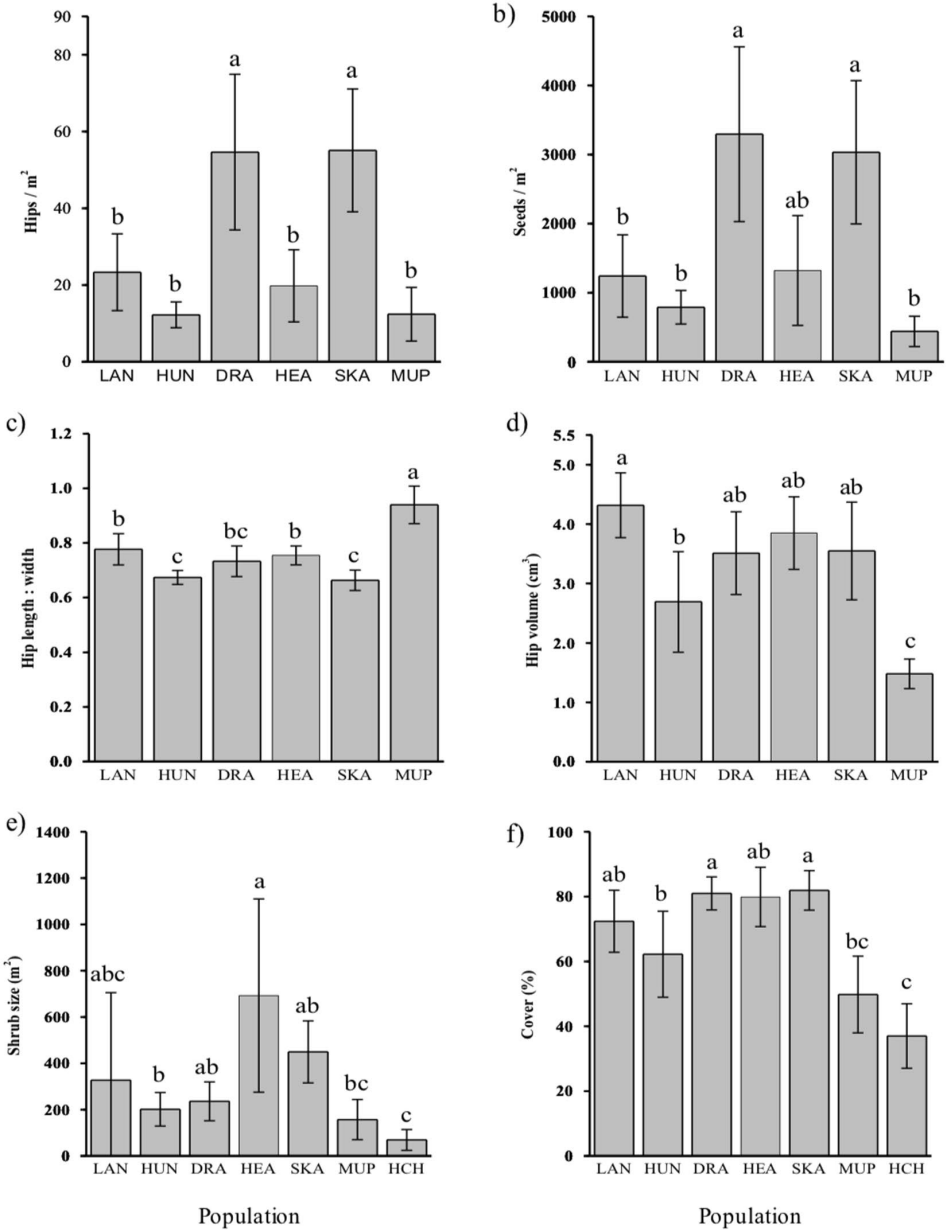
The bar plots of means with 95% confidence intervals (mean ± 1.96 SE) indicating trait differences among populations. (**a**) Hips/m^2^, (**b**) Seeds/m^2^, (**c**) Hip length: width, (**d**) Hip volume (cm^3^), (**e**) Shrub size (m^2^), and (**f**) Cover (%). The introduced European populations are Langeoog LAN, Hundige HUN, Dragør DRA, Heatherhill HEA, and Skanor SKA, while native Chinese populations are Muping MUP and Hunchun HCH. There were no significant differences between bars marked by the same letter.

At a population scale, the native Muping population produced fewer hips and lower seed production/m^2^ compared to Dragør and Skanor, but there was no significant difference in hip and seed production between Muping, Hundige and Heatherhill. Muping had significantly larger hip length:width (longer and thinner shaped hips) and smaller hip volume compared to the sampled invasive populations. The native Hunchun population had lower cover compared to the sampled invasive populations, with Muping displaying intermediate values (Fig. 1).

### Variation in reproductive traits among invasive populations

There was clear variation in reproductive traits across the five invasive populations. Dragør and Skanor showed higher hips/m^2^ and seeds/m^2^, while seed/m^2^ of Heatherhill was in the middle (Fig. 1a,b). Hundige and Skanor produced flatter hips with smaller hip length: width compared to Langeoog and Heatherhill, while hip length: width of Dragør was intermediate (Fig. 1c). Langeoog showed larger hip volume compared to Hundige, and the hip volumes of the other three populations were intermediate (Fig. 1d).

### Variation in growth traits among invasive populations

The five invasive European populations also differed in growth traits. Heatherhill shrubs were larger compared to Hundige, while the shrubs of the other populations were in the middle (Fig. 1e). Dragør and Skanor had greater coverage compared to Hundige, while the coverage of Langeoog and Heatherhill were intermediate (Fig. 1f). Dragør (ca. 111 cm ± SD 22) and Langeoog (ca. 110 cm ± SD 40) shrubs were significantly higher than those in Hundige (ca. 77 cm ± SD 21) and Skanor (ca. 71 cm ± SD 2), with Heatherthill in between (ca. 97 cm ± SD 18).

## Discussion

Many previous studies have explored performance differences between native and invasive populations across several species^3^. However, exploring the performance differences among invasive populations is understudied^4,5^, despite this information being important to determine and predict which populations are likely to be most invasive. The relationship between invasiveness and reproduction and growth traits has been established in previous studies^1,3,32^.

Here we show that the sampled invasive European population of *Rosa rugosa* had significantly higher performance than the sampled native Chinese population at a regional scale. However, these trends were not consistent among the five invasive European populations, which displayed significant variation. For example, the invasive Hundige population of Denmark had lowest average hip production, seed production, hip volume, shrub size and cover. It had similar mean trait values in hip and seed production and shrub size to the Chinese Muping population. The four other European populations (Dragør and Heatherhill in Denmark, Skanor in Sweden, Langeoog in Germany) were characterized by dramatically increased reproduction and/or growth.

Though our population sampling unbalanced (more invasive than native populations), the trait differences among invasive populations were tested while controlling for region effects, thus the results were statistically reliable. However, our study would be improved by surveying additional native populations, across its whole native range and to balance the design. Further, controlled experiments in field and/or common gardens would benefit the teasing apart of genetic vs. environment effects on these traits. However, sampling more native populations may prove difficult with the rapid rate of decline of these native populations in China. Despite these limitations, our results are informative and helpful to understand the invasiveness of this species.

Our results suggest that management of *R. rugosa* in Europe should focus on limiting the spread of high performing populations in reproduction and growth traits, but we would also emphasize that there is potential to eliminate the poor performing Hundige population. However, the management of invasive *R. rugosa* populations and the possible effects of specific management strategies on population dynamics are complex. Thus, future studies on trait comparisons among invasive populations with different management regimes should be considered in the future.

### Regional differences

At a regional scale, the sampled invasive *Rosa rugosa* in Europe performed significantly better than the native China samples. This is consistent with the behaviour of many other invasive plant species. For example, populations of the invasive shrub *Buddleja davidii* in Germany had increased growth and reproduction over its native populations in Southwest China^5^. The invasive *R. rubiginosa* populations in south Argentina had increased hip and seed production in comparison to its native populations in Spain and Germany^16^. Seeds/m^2^ of *R. rugosa* was 600–1300 in Russia^33^. In our study, the mean seed production of *R. rugosa* was considerably higher in Northwest Europe (1862/m^2^) and lower (440/m^2^) in China, which suggests increased fitness and invasion success of the species in Europe.

Similarly, greater mean values of performance traits in invasive species were found in 20 invasive-native species pairs^32^, and the importance of mean trait values for plant invasiveness was recognized in a multivariate framework^34^. Thus, the greater mean values of fitness related growth and reproductive performance traits of the sampled invasive *R. rugosa* populations in Northwest Europe could be new evidence of the invasion success of the species, combining its expansion in variable coastal habitats^26,35,36^. However, the genetic and ecological mechanisms underlying the increased performances of *R. rugosa* in its invasive range remain unclear, and further genetic and ecological studies are required to uncover the drivers of invasiveness (e.g. reciprocal transplant or common garden trials).

### Variation among invasive populations

The five invasive *R. rugosa* populations showed significantly different levels of growth and reproductive traits. Populations of Langeoog, Germany and Heatherhill, Denmark, which dominate vegetation on dunes, were more vigorous growers than the other populations we sampled. However, populations of Dragør, Denmark and Skanor, Sweden, which dominate on beaches, had greater reproductive output. The ecological context of the population in Hundige (Denmark) was different, as it was competing with tall grasses and *Hippophaë rhamnoides* on a manmade beach, and most likely as a consequence of this increased competition, it performed the worst of our studied invasive populations. Our study design does not allow us to tease apart genetic from environment effects on performance, but our data do indicate some populations of *R. rug*osa that are more invasive. Similar performance trait differences among invasive populations were also found in *R. rubiginosa*, in which its invasive populations in south Argentina perform better than ones in central Argentina^16^.

*R. rugosa* in Dragør and Skanor exhibited the highest mean hip and seed production, while the shrubs in Langeoog, Heatherhill and Hundige had less hip seed production. Increased sexual reproduction is a trait that commonly indicates invasiveness for many introduced species^9^, but the underlying mechanism may related to specific genotypes or environmental contexts (or both). We found *R. rugosa* shrubs in Dragør and Skanor had flatter hips with smaller hip length: width, and since hip shape is known to have a strong genetic basis^37,38^, this variation might indicate specific genotype differences that relate to invasiveness.

The Langeoog and Heatherhill populations had greater mean shrub size than the other European populations, and might be affected by habitat differences. Dragør, Skanor and Hundige populations are on Baltic Sea beaches, where the soil is salty and nutrient poor. Langeoog and Heatherhill populations were on the dunes, where the soil is more fertile and less salty^24^. In accordance with Grime^39^, in harsh environmental conditions (e.g. salty Baltic Sea beach conditions), plants are more stressed and likely to grow less vigorously but produce more seeds and fruits. Also, other invasive species, such as invasive dayflowers (*Commelinaceae*), have been shown to grow more vigorously in nutrient rich habitats^8^.

## Conclusions

Most studies on invasive plant performance compare invasive with native populations, yet performance differentiation among introduced populations is crucial information for managing invasive species. Variation in our sampled introduced *R. rugosa* populations is of practical concern, as in our case, we observed considerable variation in shrub growth vigour and reproductive output among the five invasive populations we studied. Though the genetic and environmental factors driving performance differentiation could not be disentangled with our design^40^, our findings do shed light on the identification of potentially more invasive *R. rugosa* populations and invasiveness indicating traits, which are likely to be importance to land managers. Determining invasiveness related traits and their variation among populations are crucial components of invasive species biology, which help understand adaptive evolution of invasive species and can be used to improve the effectiveness of invasive species management.

## Methods

### Study sites

**Figure 2:**
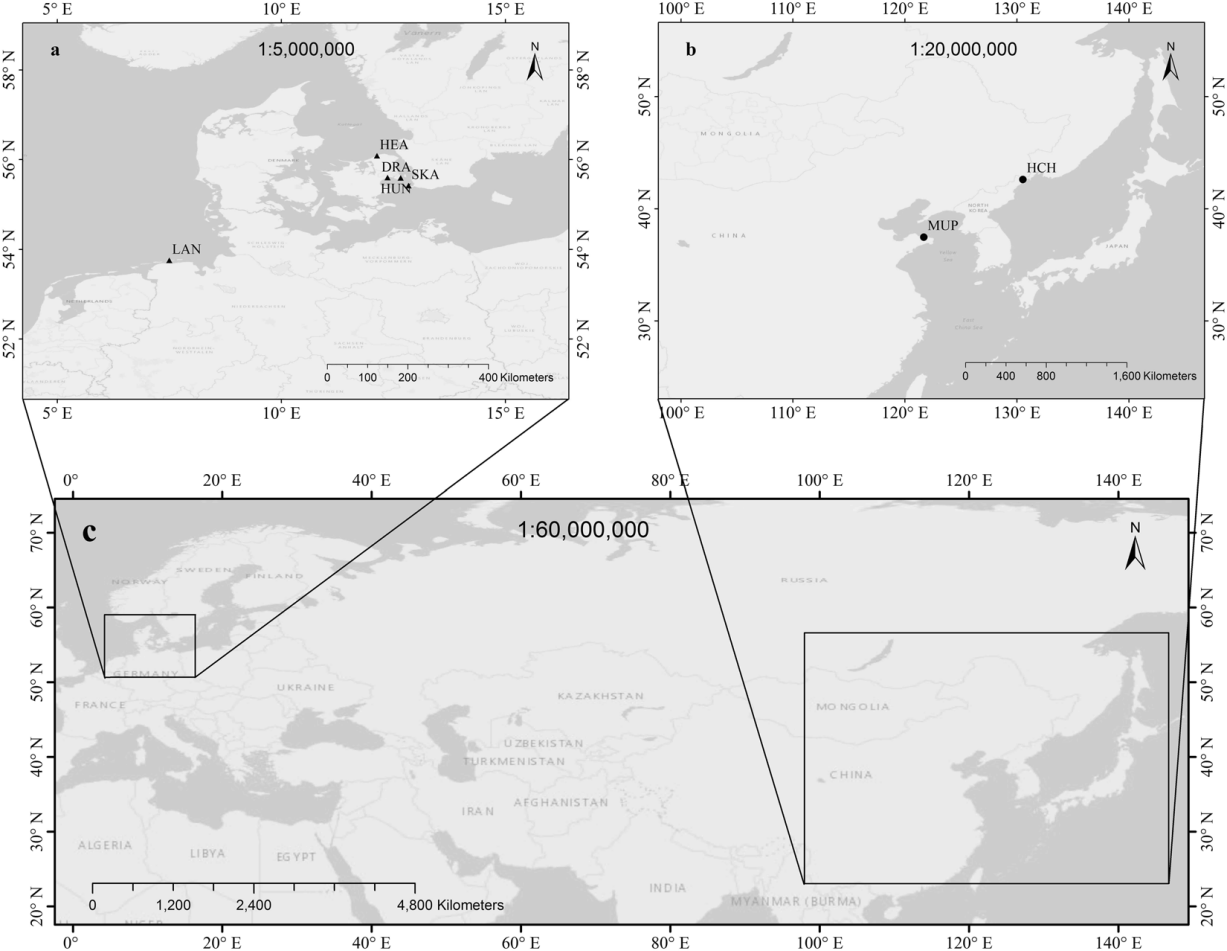
Maps of the sampling sites and investigated regions. The solid black triangles in map (**a**) indicate the sampling sites of five introduced populations in Europe including LAN Langeoog, HUN Hundige, DRA Dragor, HEA Heatherhill, and SKA Skanor, the solid black dots in map (**b**) indicate the sampling sites of two native populations in China including MUP Muping and HCH Hunchun, and the two rectangles in map (**c**) show the two investigated regions in Eurasia.

We sampled five invasive populations from northwest Europe and two native populations from China in October 2012 (Fig. 2). All populations occurred on sandy soil of different habitats, including sandy beaches, dunes or sandy cliffs (Table 3). All invasive populations had similar mean annual temperature (ca. 8 °C) and mean annual precipitation (ca. 600–800 mm; www.en.climate-data.org). The two native populations have similar mean annual precipitation (ca. 600–700 mm), but vary in mean annual temperature (Hunchun ca. 5.9 °C, Muping ca. 12.7 °C). The invasive populations receive relatively even rainfall across the year, whereas both native populations have more seasonal rainfall, with a wetter summer (ca. 300–400 mm from June to August) and a drier winter (ca. 15–40 mm from December to February).

**Table 3:** Location, sample size and habitat of each investigated *Rosa rugosa* population.

Population	Abbreviation	n	Habitat
**Introduced populations**			
Langeoog, Germany	LAN	15	Manmade sand dunes, North Sea
Hundige, Denmark*	HUN	13	Manmade sand beach, Baltic Sea
Dragor, Denmark*	DRA	11	Sandy beach, Baltic Sea
Heatherhill, Denmark*	HEA	10	Sandy cliff facing the Kattegat
Scanor, Sweden*	SKA	12	Sandy beach, Baltic Sea
**Total**		61	
**Native populations**			
Muping, China	MUP	10	Sand beach, Bohai Sea
Hunchun, China	HCH	10	Sand dunes, Tumenjiang Valley
**Total**		20	

Five naturalized introduced populations from Northwest Europe and two native populations from China were sampled. The geographic locations of all seven populations are shown in Fig. 2.

*Populations with partial mowing management.

The five invasive populations included Langeoog, a North Sea island (Germany) and part of the Wadden Sea National Park. This population is on sand dunes, is protected from mowing, cutting and digging, but biking and hiking on nearby paths are allowed. The second population was Heatherhill (Denmark), which is on sandy cliffs northwest of Copenhagen. Mowing of some large *R. rugosa* shrubs is allowed. The last three populations were Hundige (Denmark), Dragør (Denmark) and Scanor (Sweden), which were each located on sandy beaches on the Baltic Sea where mowing management is applied to some of the shrubs to keep the beaches open. In most cases, invasive *R. rugosa* in Europe frequently develops dominant stands, with occasional coexisting shrubs (e.g. *Calluna vulgaris*, *Empetrum nigrum* ssp. *nigrum, Hippophaë rhamnoides)* and tall grasses (e.g. *Ammophila arenaria, Leymus arenarius*).

We sampled two native populations from China, one from Hunchun and the other from Muping. These populations were in Hunchun and Jiaodong nature reserves, respectively. Neither population is managed by mowing/cutting. However, people in Hunchun harvest *R. rugosa* flowers, which can drastically reduce the number of hips (accessory fruit with achenes and seeds) in autumn. The co-dominant shrubs *Sophora flavescens* and *Vitex rotundifolia* and herbs *Artemisia argyi*, *Carex kobomugi*, *Ischaemum bartatum*, and *Vicia japonica* frequently occur at the sites of both native populations.

### Sampling strategy

We used a nested sampling design, with three spatial scales: regional (China vs. Northwest Europe), population nested within region (two populations from China and five populations from Northwest Europe), and individual shrub nested within population. Within each population, ten to fifteen shrubs were randomly sampled for trait measurements (Table 3). Sampled shrubs were not nearest neighbours and were more than 10 m apart to avoid sampling clones.

We employed different sampling methods to measure traits between the native and invasive populations because the shoots of the native shrubs were sparser and more scattered than the invasive shrubs. To measure traits in the introduced populations we used two 1 × 1 m quadrates across the shrub canopy, one in the centre and one on the edge of the shrub. For the native populations, we used four to twelve 0.5 × 0.5 m quadrates per shrub through the centre of the shrub (quadrate number depended on the size of the canopy).

We measured eight performance traits per shrub. Five traits were related to reproductive output: (1) hips per square meter in sampled quadrates (hips/m^2^); (2) seeds per hip for 10 hips per shrub; (3) seeds per square meter (seeds/m^2^) calculated from (1) × (2); (4) mean hip volume for the same 10 hips per shrub (hip volume = π(hip width/2)^2^ × (hip length/2); and (5) ratio of hip length to width (hip length: width) for the same 10 hips per shrub. Three growth vigour traits were also estimated: (6) shrub size (estimated by multiplying shrub length and width; m^2^); (7) shrub canopy cover in sampled quadrates (% cover); and (8) shrub height in sampled quadrates (cm).

### Data analysis

The effects of region and population on performance traits were estimated with nested general linear models in R v. 3.4.3^41^. Region was treated as a fixed effect, and population was treated as a fixed effect nested within region. Seeds/m^2^ and shrub size were square root transformed to increase normality of residuals and reduce heteroscedasticity.
